# Supplementary material for: Attenuating RNA Viruses with Expanded Genetic Codes to Evoke Adjustable Immune Response in PylRS-tRNACUAPyl Transgenic Mice
Source: Vaccines (Basel). 2023 Oct 17;11(10):1606. doi: 10.3390/vaccines11101606 (PMC10610612; doi:10.3390/vaccines11101606)
Supplement: Supplementary file 1 [file vaccines-11-01606-s001.zip › vaccines-2559431-Supplemantary Materials.pdf]

Supporting Information for:

Attenuating RNA Viruses with Expanded Genetic Codes to Evoke Adjustable Immune Response in PylRS-$tRNA^{Pyl}_{CUA}$ Transgenic Mice

Zhetao Zheng[#], Xuesheng Wu[#], Yu Wang, Xu Yang, Hongmin Chen, Yuxuan Shen, Yuelin Yang, Qing Xia*

State Key Laboratory of Natural and Biomimetic Drugs, Department of Chemical Biology, School of Pharmaceutical Sciences, Peking University, Beijing 100191, China

[#] These authors contributed equally to this work.

* Email: xqing@hsc.pku.edu.cn

**Experimental Section**	
**Figure S1**	Establishment of the VeroE6-$tRNA^{pyl}$/PylRS/GFP^{39TAG} stable cell line.
**Figure S2**	Representative GO terms and KEGG enrichment analysis of HEK293T+NAEK cells and the engineered packaging systems compared with HEK293T cells.
**Figure S3**	Whole-transcriptome analysis of Vero cells and the engineered production systems.
**Figure S4**	CPE of EV71-NAEK virus harboring an amber codon at VP1–VP4, 2A–2C, and 3A–3D by random-site selection.
**Figure S5**	CPE imaging of EV71-NAEK strains produced by the NAEK incorporation system in the presence or absence of NAEK.
**Figure S6**	Guideline for and validation of NAEK-controllable virus design for other single-stranded RNA (ssRNA) viruses.
**Figure S7**	Structural illustration of 35 residues on EV71 3C/D.
**Figure S8**	Generation of UAA-controllable Enteroviruses.
**Figure S9**	PCR analysis of mouse tail DNA from six descendants of transgenic mice crossed with wild-type mice.
**Figure S10**	Bioavailability of NAEK in mice.
**Figure S11**	Original western blot images.

Experimental Section

Cell culture

The human embryonic kidney 293T (HEK293T, CRL-11268) cells and Vero-E6 (CRL-1586) cells were obtained from ATCC. HEK293T cells were cultured in Dulbecco's modified Eagle's medium (DMEM) (Life Technologies) supplemented with 10% fetal bovine serum (FBS) (Gibco), 100 IU/mL penicillin and 100 μg/mL streptomycin (Invitrogen). Vero-E6 cells were cultured in Eagle's Minimum Essential Medium (MEM) (Life Technologies) with the same supplements. The cells were cultured at 37 °C and under 5% CO_2, and passaged upon reaching 80%-90% confluence. Authentication and test for the free of mycoplasma were performed by our lab.

Virus and vaccine

Enterovirus 71 AH/08/06 (Genbank: HQ611148.1, genotype C4) was used as a study model. The enterovirus type 71 vaccine (inactivated) and the EV71_SD059 strain lethal strain was provided by Sinovac Biotech (Beijing).

Plasmid construction

The infectious full-length cDNA clone of EV71-A12 genome was kindly provided by Professor Wu Zhong from the Academy of Military Medical Sciences. Mutant plasmids (EV71-A12-3C-TAG, EV71-A12-3D-TAG) containing amber codons within the open reading frame were generated by the QuikChange method (SBS Genetech) and confirmed by gene sequencing (Tsing ke, Beijing). The infectious full-length cDNA clone of CA10, CA16 and EVD68 were kindly provided by Professor Ningshao Xia from Xiamen University and were substituted with the same SP6-promoter transcription vector as EV71-A12. The Nucleocapsid protein sequence from SARS-CoV-2 (isolate Wuhan-Hu-1) was synthesized in BGI and fused to TagBFP gene by Gibson assembly. The *Methanosarcina mazei* (Mm) pyrrolysyl tRNA synthetase/$tRNA_{CUA}$ pair (MmPylRS/$tRNA^{MmPylRS}_{CUA}$) for site-specific incorporation of Nε-2-azidoethyloxycarbonyl-L-lysine (NAEK) was synthesized as previously reported[1]. The Tol2 transposon systems were purchased from Biocytogen (Beijing, China). The MmPylRS gene driven by CAG promoter was cloned into the Tol2 transposon vector to obtain Tol2-pylRS plasmid. The GFP gene with an amber codon

introduced at residue position 39 was expressed under a CMV promoter and cloned into the Tol2-pylRS plasmid to obtain Tol2-pylRS-GFP^{39TAG} plasmid. The pcDNA 3.1 vectors carrying 6 $tRNA_{CUA}^{MmPylRS}$ copies driven by human H1, human U6 and human 7sk promoters respectively were preserved in our lab. Then it was used as template to arrive the 12 tRNA $_{CUA}^{MmPylRS}$ copies which were then cloned into the Tol2-pylRS-GFP^{39TAG} plasmid via Gibson assembly to obtain the Tol2-12tRNA^{pyl}-pylRS-GFP^{39TAG} plasmid, used for NAEK-Vero stable cell line construction subsequently. All plasmids used for transfection were amplified using a Maxiprep kit (Promega) according to the manufacturer's instructions.

Establishment of NAEK-293T stable cell line

HEK293T cells were used for lentiviral vector packaging and transduction. The cells were cultured in DMEM medium (Macgene, without sodium pyruvate), supplemented with 10% FBS (PAA), and 1 mM nonessential amid acids (Gibco) in 6-well plates until sub-confluent. Then, cells were co-transfected with 0.72 μg of pSD31 transfer plasmid, 0.64 μg of pRSV, 0.32 μg of pMD2G-VSVG and 0.32 μg of pRRE using the transfection reagent Megatran1.0 (Origene). After 6 h, the transfection medium was replaced by DMEM medium containing 3% FBS and 1 mM nonessential amid acids. 48 h post-infection, the lentivirus-containing supernatant was harvested and filtered through a 0.45 μm filter. The resultant dual lentiviruses pSD31-PylRS and pSD31-GFP^{39TAG} were used to integrate MmPylRS and the GFP^{39TAG} gene into the genome of HEK293T cells. Experiments for stable lentiviral transduction were carried out as follows: HEK293T cells were seeded in a 6-well plate and transduced with lentiviral filtrates in the presence of 8 μg/mL polybrene 24 h later. Then, selection was performed under the pressure of 600 ng/mL puromycin and 200 μg/mL hygromycin until parental cells completely died. The resultant stably transduced HEK293T-PylRS/GFP^{39TAG} cells were transfected with linearized bjmu-12$tRNA_{CUA}^{MmPyl}$-zeo plasmid DNA and cultured under the pressure of 200 μg/mL Zeocin until parental cells completely died. In the presence of UAA, the stably transfected cells were then sorted by fluorescence-activated cell sorting (FACS) according to the GFP phenotype and verified by their dependence on UAA for GFP expression.

Establishment of NAEK-Vero stable cell line

For electroporation, Vero-E6 cells were resuspended in Opti-MEM and counted, and 1×10^6 cells were mixed with 15 μg of linearized Tol2-12tRNA^{pyl}-pylRS-GFP^{39TAG} plasmid in medium cup (EC002S). The electroporation was performed using a CUY21EDIT square-wave electropulser (Nepa Gene Co., Ltd) under the following parameters: 150 V, poring pulse (5 ms with 50 ms intervals); 20 V, transfer pulse (50 ms with 50 intervals). After electroporation, the cells were immediately moved from the cup and plated on three 10-cm diameter tissue culture dishes in complete medium with 20% FBS. After 24 h, the plates were washed twice with Opti-MEM and re-fed with complete medium (10% FBS). To obtain stably transfected Vero, the cells were first incubated in complete medium with 20% FBS for 72 h and then in complete medium with 4 mM NAEK and 2 μg/ml puromycin. Nearly after 13 days, the Vero cells containing the transfected gene exhibited a phenotype to form clones. Then the pooled clones were cultured under puromycin pressure. In 15 days, about 80% of the Vero cells expressed GFP fluorescence, indicating MmPylRS/tRNA$^{MmPylRS}_{CUA}$ gene was functional to read-through the amber codon introduced in GFP. The cells were sorted by FACS to obtain the monoclonal stable cell line.

Generation of wild type EV71 viruses and UAA-controllable EV71 viruses

The wild type EV71 generated from reverse genetic system was performed as previous report[2]. 2×10^5 cells per well from the HEK293T-tRNA/PylRS/GFP^{39TAG}, HEK293T-6GlnstRNA/GFP^{39TAG} and HEK293T-3CD/GFP cell lines were seeded into six well plates in DMEM supplemented with 10% FBS for 24 h before transfection. Then the wild type EV71 plasmid was linearized by MluI restriction enzyme digestion. The 1 μg linearized plasmid was serving as a template for *in vitro* transcription (SP6, Promega), and the products was then transfected into cells with Lipofectamine 2000 (Invitrogen). Six hours later, the medium containing the mixture of mRNA and lipofectamine 2000 reagent was replaced with DMEM supplemented with 1% FBS. The cells were further incubated at 37 °C in 5% CO_2 until >90% CPE was observed, and the supernatant containing the generated virus was harvested and centrifuged at 1000 g for 10 min to remove contaminating cells.

To generate EV71-NAEK virus, an almost identical procedure was carried out, with the following changes: The plasmid expressing wild type viral RNA was replaced by the

corresponding mutant plasmid, and the medium was further supplemented with 1 mM NAEK when the virus was packaged by UAA system for viral packaging. To identify the UAA dependent viral strains, a parallel packaging experiment was conducted without UAA supplement.

Western blot analysis

HEK293T cells were lysed in RIPA lysis buffer (Applygen) supplemented with complete protease inhibitor cocktail (Roche) 48 h after transfection, and cell debris was removed by centrifugation. Tissue was then broken with a homogenizer and removed by centrifugation at 4 °C. Protein was extracted with lysis buffer and quantified by BCA assay (Thermo). A total of 100 μg protein from each sample was boiled with loading buffer, separated on 4%-12% NuPAGE (Invitrogen), and then electroblotted onto a polyvinylidenedifluoride membrane. The membrane was blocked with 5% (v/v) nonfat milk in TBST (50 mM Tris-HCl, 150 mM NaCl, and 0.02% Tween-20, pH 7.5) at room temperature for 1 h and then incubated with rabbit/mouse polyclonal antibodies overnight at 4 °C. Anti-3D antibodies (1:500, ab15277, Abcam), anti-N protein antibodies (1:500, ab7164, Abcam), anti-GFP antibodies (1:3000, 12715-1-AP, Proteintech), anti-PylRS antibodies (1:3000, sc-365062, Santa Cruz Biotechnology, to detect Myc-tagged PylRS) were diluted in TBST containing 5% (v/v) of defatted milk. The membranes were rinsed three times with TBST and then incubated with horseradish peroxidase-conjugated goat anti-rabbit/mouse IgG (1:3000) at room temperature for 1 h. After extensive washing, the protein bands were developed using an enhanced chemiluminescent detection kit (Millipore). The optical bands were visualized in a Fuji Las-3000 dark box (FujiFilm), and band densities were quantified using Quantity One Analysis software. All western blot experiments were done for at least three times in parallel and the representative results were reported.

Flow cytometry

Until transfected cells were treated with antibiotics to form clones, cells were dissociated into single cell using trypsin/EDTA and analyzed on BD FACSAriaTM (BD Biosciences) with the appropriate filter settings (488 nm coherent sapphire laser for GFP excitation). The front and side scatters were used to identify intact cells and mean

background fluorescence from untransfected cells was subtracted from the measured signal. GFP positive cells were harvested for further validation of integrated genes.

Virus growth curve analysis

To determine *in vitro* virus growth rates, triplicate wells of confluent transgenic cells, HEK293T-tRNA/PylRS/GFP^{39TAG}, HEK293T-6stRNA^{Gln}/GFP^{39TAG} and HEK293T-3CD/GFP, (6-well plate format, 10^6 cells/well) were infected at a MOI of 0.1. After 1 h of virus adsorption at 37 °C, cells were washed and overlaid with DMEM supplemented with 1% FBS, and additional treatment with 1 mM NAEK in UAA system. At the indicated times post-infection (on day 1, 2, 3, 4, 5, 6 and 7), the cell supernatants were collected and viral titers were determined by the qRT-PCR as described above.

Enterovirus 71 quantification by 50% tissue culture infective dose

For quantifying all viruses' stocks, the 50% tissue culture infective dose ($TCID_{50}$/mL) titers were determined. In brief, 5×10^4 Vero-E6 cells (or NAEK-Vero cells for EV71-NAEK quantification) were seeded in 96-well plates the day before infection. The virus samples were serially diluted with DMEM containing 1% FBS (10^3 to 10^{10}) and then each of dilution was added in wells separately. The plates were incubated at 37 °C in 5% CO_2 for 2-5 days. The cytopathic effect (CPE) was observed under a microscope and virus titer was determined using the Reed-Münch endpoint calculation method (http://www.fao.org/3/AC802E/ac802e0w.htm).

Generation of MmPylRS/tRNAMm$^{Pyl}_{CUA}$ transgenic mice

Methods to generation of transgenic mice are previously described[1,3,4]. In brief, we chose the ROSA26 site of the autosome and utilized *Streptococcus pyogenes* Cas9 (SpCas9) and designed sgRNA to cut the targeted genome, which was then recombined with the transgene of CAG pro-MmPylRS-IRES-EGFP^{39TAG}-WPRE-PA-(7SK-tRNA$^{MmPyl}_{CUA}$)$_4$ in the pronucleus of fertilized eggs derived from parental mice (C57BL/6♂ x C57BL/6♀). Subsequently, we performed embryo transplantation into surrogate ICR females to breed the F0 generation. Three weeks after birth, the tail DNA of all the F0 generation mice was extracted for PCR analysis to identify the positive mice, which were further crossed with wild type C57BL/6 mice (♀) to produce the next generation.

Animal experiments

All animal experiments were performed in accordance with the guidelines of the Institutional Animal Care and Use Committee of the Peking University (approval number: LA2021325). Briefly, groups of 4-week old female BALB/c mice (n=5) were inocubated by intraperitoneal injection with EV71-NAEK virus, Sinovic EV71 vaccine (positive control) and PBS (negative control) respectively to test the immunogenicity, efficacy and safety. All Mice were monitored daily for body weight and survival rate. Two weeks post-inoculation, five mice of each group were sacrificed. The sera were used to test their immunogenicity by ELISA and organs (e.g., brain, intestine and skeletal muscle) were collected to detect viral titer by qRT-PCR.

For protective efficacy study, five BALB/c mice of 4-week old in each group were immunized for twice (0 and 14 days) with the same dose. Two weeks after the second immunization, the female mice were paired with male mice until the born of neonatal mice. Four-day old neonatal mice were intraperitoneally injected with 10 LD_{50} of EV71_SD059 strain (lethal strain, Sinovac Biotech, Ltd, Beijing). The body weight and survival rate of mice were observed for 7-14 days until the mice returned to normal.

For *in vivo* UAA-controllable infection investigation, eight transgenic mice of 4-week old in each group were intraperitoneally injected with EV71-3D-E105NAEK, wild type EV71 virus and PBS after genotype identification by PCR analysis. All mice were intraperitoneally injected with different doses of NAEK and monitored daily for body weight and survival rate. Three days after infection, three mice of each group were sacrificed to detect viral titer by qRT-PCR and pathological changes by immunofluorescence staining and histological analysis. Other five mice of each group were sacrificed in two weeks to collect their sera for neutralizing antibodies detection.

Quantitative Real-Time PCR analysis

Total RNA harvested from cells or mice tissues infected with distinct viruses or vaccine was extracted by using Trizol reagent (Invitrogen, CA, USA). RNA (1 μg) was subjected to RT-PCR in accordance with the protocol provided by Promega. The transcripts were quantitated and normalized to the internal GAPDH control. The PCR conditions were 1

cycle at 95 °C for 5 min, followed by 40 cycles at 95 °C for 15 s, 60 °C for 1 min, and 1 cycle at 95 °C for 15 s, 60 °C for 15 s, 95 °C for 15 s. The results were calculated using the $2^{-\triangle\triangle CT}$ method according to the GoTaq qPCR Master Mix (Promega) manufacturer's specifications.

The whole transcriptome analysis

RNA sequencing was performed by Annoroad Inc. (Beijing, China). Briefly, total mRNA was purified by poly-T oligo-attached magnetic beads and fragmented. Sequencing libraries were generated using NEBNext® Ultra™ RNA Library Prep Kit for Illumina® (#E7530L, NEB, USA) following the manufacturer's recommendations and index codes were added. The libraries were sequenced on an Illumina platform and 150 bp paired-end reads were generated. The raw data were quality controlled by Q30 and the clean data was aligned to the reference genome (Ensembl hg38) using HISAT2 (v2.1.0) software. Reads Count for each gene in each sample was counted by HTSeq v0.6.0, and FPKM (Fragments Per Kilobase Millon Mapped Reads) was then calculated to estimate the expression level of genes in each sample. DESeq was used for differential gene expression analysis between two samples and Genes with $q \leqslant 0.05$ and $|log2\_ratio| \geqslant 1$ were identified as differentially expressed genes. GO and KEGG pathway enrichment analysis were performed based on differentially expressed mRNAs.

Enzyme-linked immunosorbent assay (ELISA)

IgM and anti-VP1 IgG antibody in sera were measured using ELISA. In this assay, 96-well ELISA plates (Thermo Fisher Scientific Inc., USA) were coated with 100 mM bicarbonate/carbonate buffer (pH 9.6) containing 0.03 μg/mL of recombinant proteins (VP1) from homologous wild-type viruses (Sino Biological Inc., Beijing, China) overnight at 4 °C, followed by 3% bovine serum albumin (BSA; Sigma) in PBS-0.05% Tween 20 (blocking buffer) blocking for 1 h at 37 °C. The coated plates were then incubated with serum samples for IgM and anti-VP1 IgG, and followed by incubating HRP-conjugated anti-mouse/ferret IgG antibody or HRP-conjugated anti-mouse/ferret IgM antibody (Sino Biological Inc., Beijing, China) for 1 h at 37 °C. Plates were detected with tetramethyl benzidine (TMB) substrate (Millipore, Billerica, MA, USA) and stopped after 15 min with

0.5 M H_2SO_4. Optical density (O.D.) was read at 450 nm using an ELISA reader (Microplate Reader AMR-100; Allsheng, Hangzhou, China).

Immunofluorescence

The intestine harvested from different mice were fixed in 4% PFA at room temperature for 1 hour, followed by immersing in 30% (w/v) sucrose until submersion before embedding and freezing in the Optimal Cutting Temperature (OCT) compound (Tissue-Tek). Serial 12 μm sections were obtained by cryo-sectioning of the embedded intestine tissue at -20 °C using a cryostat (Leica). Cryosections were blocked with 5% normal donkey serum (Jackson ImmunoResearch) in PBST for 30 min. The sections were incubated with anti-3D antibody (1:2000, GTX630193, GeneTex) diluted in blocking buffer at 4 °C overnight. The slides were subsequently incubated with secondary goat anti-rabbit IgG Alexa Fluor 594 (1:400, LifeTech) at room temperature for 1 h. The slides were stained with 0.5 μg/mL Hoechst and mounted in mounting media. Stained sections were photographed under a Nikon Ti-S microscope, and the dystrophin-positive cells were analyzed.

Histological analysis

The tissues of different mice were isolated and fixed in 4% PFA solution for two days, sequentially dehydrated with 70%, 95% and 100% ethanol, and defatted with xylene for 2 h before being embedded in paraffin. The 10-μm-thick section was cut and subjected to hematoxylin and eosin staining (H&E staining). The slices were observed under optical microscopy, and the histological morphology of different mouse groups was compared.

Statistical analysis

Data are presented as mean ± SD. In most circumstances, three replicates were used for each group unless otherwise noted. The statistical significance between two groups was determined by student's t test. For three or more groups, one-way or two-way ANOVA with Tukey's multiple comparisons test was performed. Homogeneity of variances among groups was confirmed using Bartlett's test. Conformity to normal distribution was confirmed using the Kolmogorov-Smirnov test. All tests were two-sided and performed in GraphPad Prism 8. A probability of $p < 0.05$ was considered as statistically significant. For annotations, * $p<0.05$; ** $p<0.01$; *** $p<0.001$; **** $p<0.0001$. The amino acid similarity was calculated

in Maestro of the Schrodinger software. Logistic regression was performed between the virus packaging results and incorporated positions features.

Reference

1. Shi, N.; Yang, Q.; Zhang, H.; Lu, J.; Lin, H.; Yang, X.; Abulimiti, A.; Cheng, J.; Wang, Y.; Tong, L.; et al. Restoration of Dystrophin Expression in Mice by Suppressing a Nonsense Mutation through the Incorporation of Unnatural Amino Acids. *Nat Biomed Eng* **2021**, 1–12, doi:10.1038/s41551-021-00774-1.
2. Roehl, H.H.; Parsley, T.B.; Ho, T.V.; Semler, B.L. Processing of a Cellular Polypeptide by 3CD Proteinase Is Required for Poliovirus Ribonucleoprotein Complex Formation. *Journal of Virology* **1997**, *71*, 578–585, doi:10.1128/jvi.71.1.578-585.1997.
3. Chin, J.W. Expanding and Reprogramming the Genetic Code of Cells and Animals. *Annual Review of Biochemistry* **2014**, *83*, 379–408, doi:10.1146/annurev-biochem-060713-035737.
4. Chen, Y.; Ma, J.; Lu, W.; Tian, M.; Thauvin, M.; Yuan, C.; Volovitch, M.; Wang, Q.; Holst, J.; Liu, M.; et al. Heritable Expansion of the Genetic Code in Mouse and Zebrafish. *Cell Res* **2017**, *27*, 294–297, doi:10.1038/cr.2016.145.

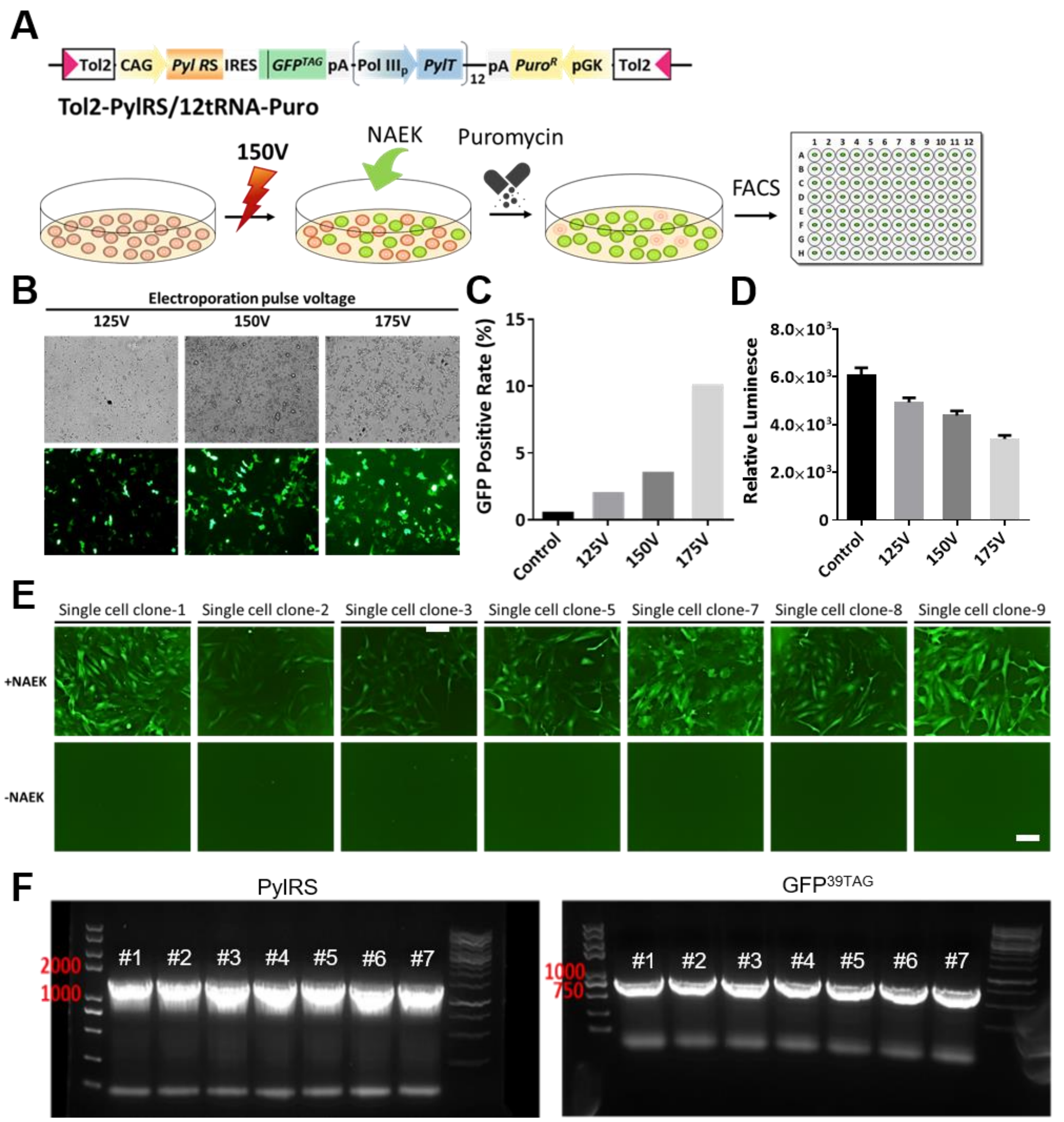

Figure S1. Establishment of the VeroE6-tRNA^{pyl}/PylRS/GFP^{39TAG} stable cell line. (A) Plasmid of the UAA incorporation system and stable cell line screening. **(B)** Comparison of transfection efficiency at different voltages. **(C, D)** Number of green fluorescent protein (GFP)–positive cells and rate of relative luminescence at different voltages. **(E)** GFP fluorescence appeared in the screened stable cell line in a NAEK-dependent manner. Scale bars, 50 μm. **(F)** PCR analysis of PylRS and GFP expression in the stable cell line.

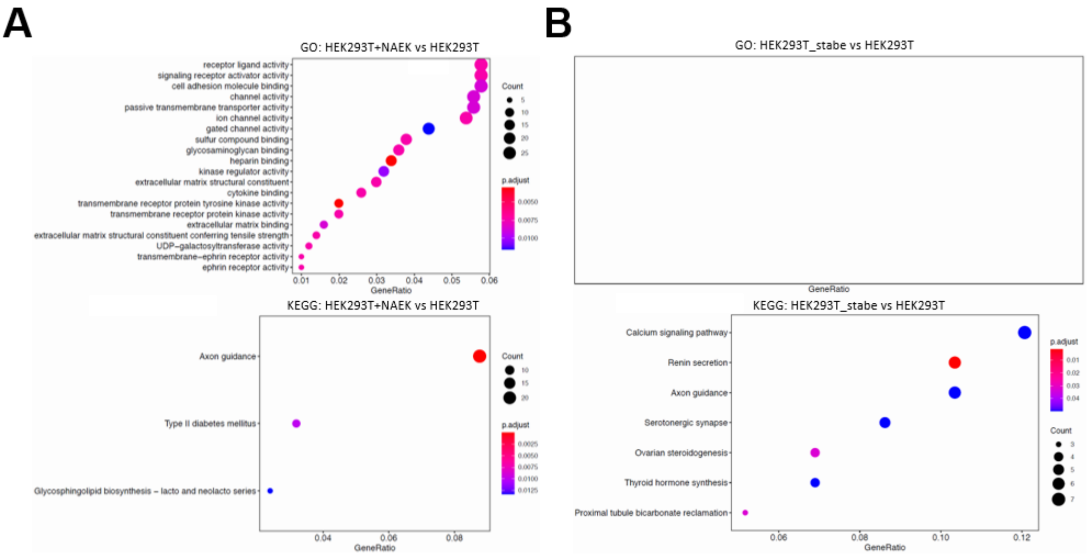

Figure S2. Representative GO terms and KEGG enrichment analysis of HEK293T+NAEK cells and the engineered packaging systems compared with HEK293T cells.

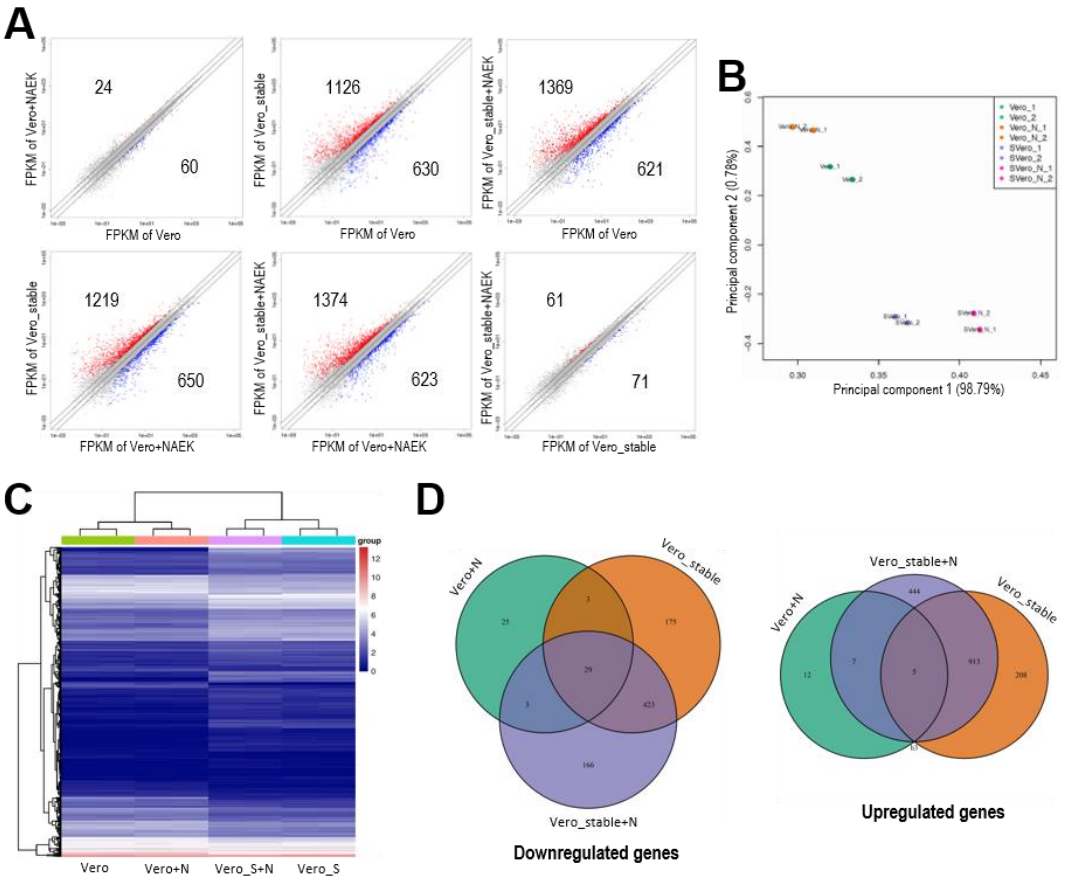

Figure S3. Whole-transcriptome analysis of Vero cells and the engineered production systems. (A) Plots show whole-transcriptome FPKMs. Red dots indicate upregulated genes; blue dots downregulated genes. Two biological replicates were used per sample. **(B)** PCA of Vero cells and the engineered production systems. **(C)** Hierarchical-clustering and heatmap analysis of DEGs in Vero cells and the engineered production systems. **(D)** Venn diagram showing significant overlap (*P* < 0.005) of DEGs between Vero cells and the engineered production systems.

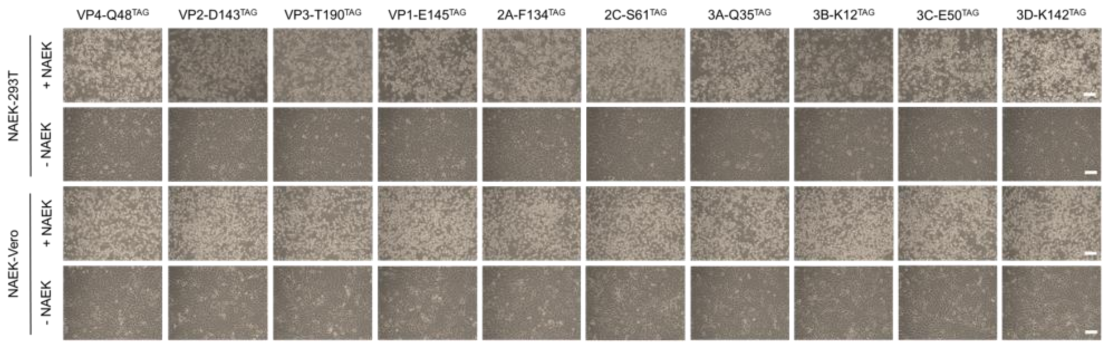

Figure S4. CPE of EV71-NAEK virus harboring an amber codon at VP1–VP4, 2A–2C, and 3A–3D by random-site selection. Scale bars, 50 μm.

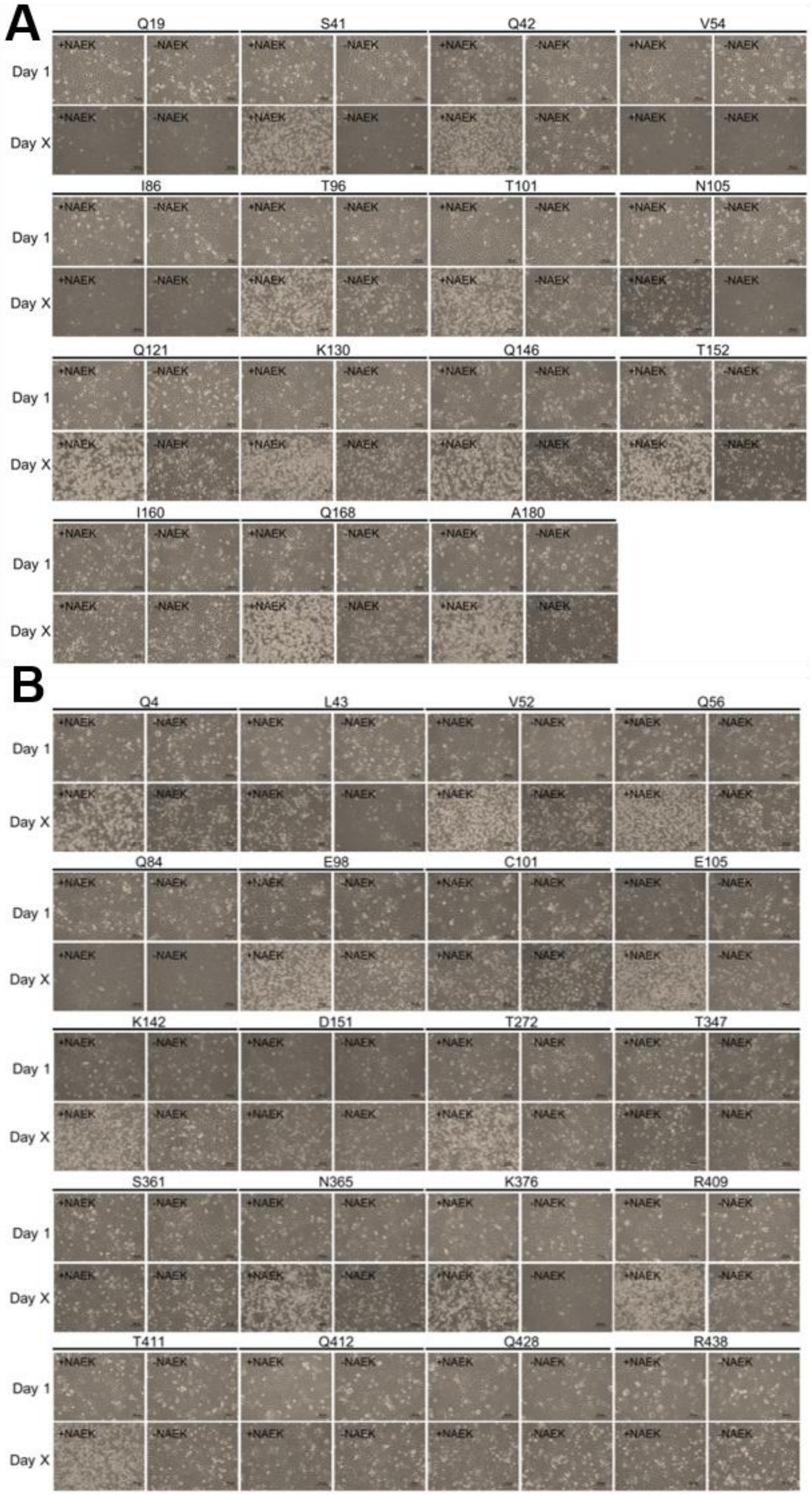
A
Q19
S41
Q42
V54
I86
T96
T101
N105
Q121
K130
Q146
T152
I160
Q168
A180
B
Q4
L43
V52
Q56
Q84
E98
C101
E105
K142
D151
T272
T347
S361
N365
K376
R409
T411
Q412
Q428
R438
Day 1
Day X
+NAEK
-NAEK

Figure S5. CPE imaging of EV71-NAEK strains produced by the NAEK incorporation system in the presence or absence of NAEK. Day X represents days required to attain ~100% CPE (n = 3). **(A)** Strains with NAEK incorporated into 3C protein. **(B)** Strains with NAEK incorporated into 3D protein.

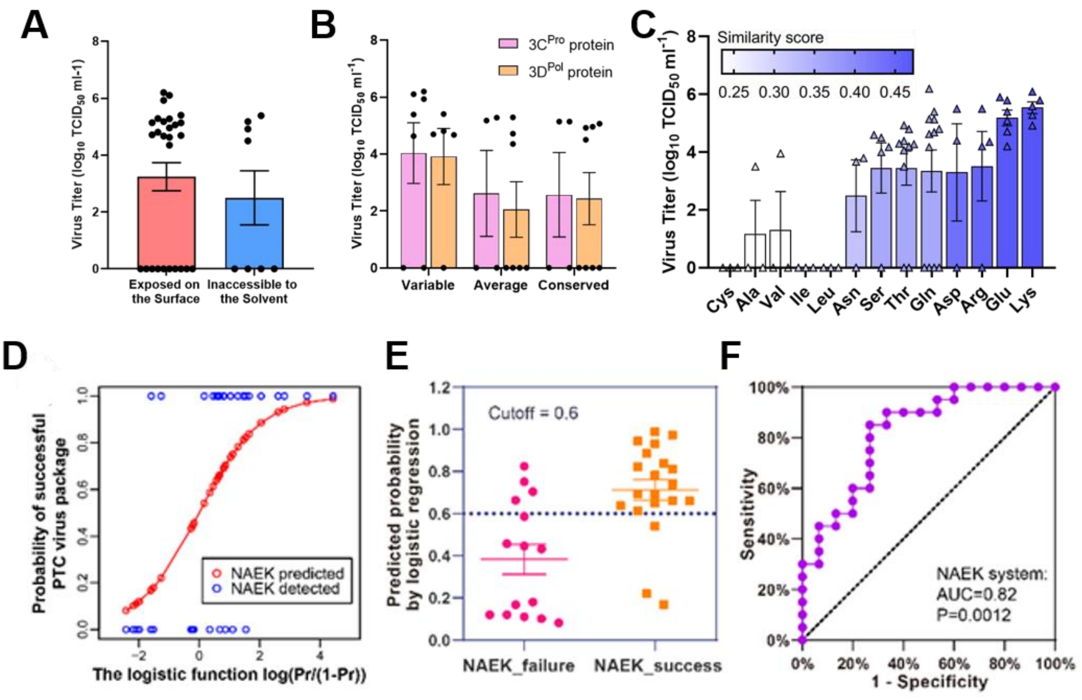

Figure S6. Guideline for and validation of NAEK-controllable virus design for other single-stranded RNA (ssRNA) viruses. (A–C) Relationship between viral titers and residue location, and conservation or similarity of amino acids. **(D)** Workflow for the guideline for and validation of controllable virus design. **(E–G)** Logistic regression of viral packaging by NAEK, based on the information of the 35 environmentally verified positions in EV71. Blue circles indicate the detected probability (0 or 1) of successful viral packaging, red circles the predicted probability thereof. Four factors were selected for logistic regression for function and related estimates. Predicted probability of success or failure per group was also plotted (mean ± standard error of the mean [SEM]), and receiver operating characteristic (ROC) curves are shown (area under the curve [AUC] > 0.8).

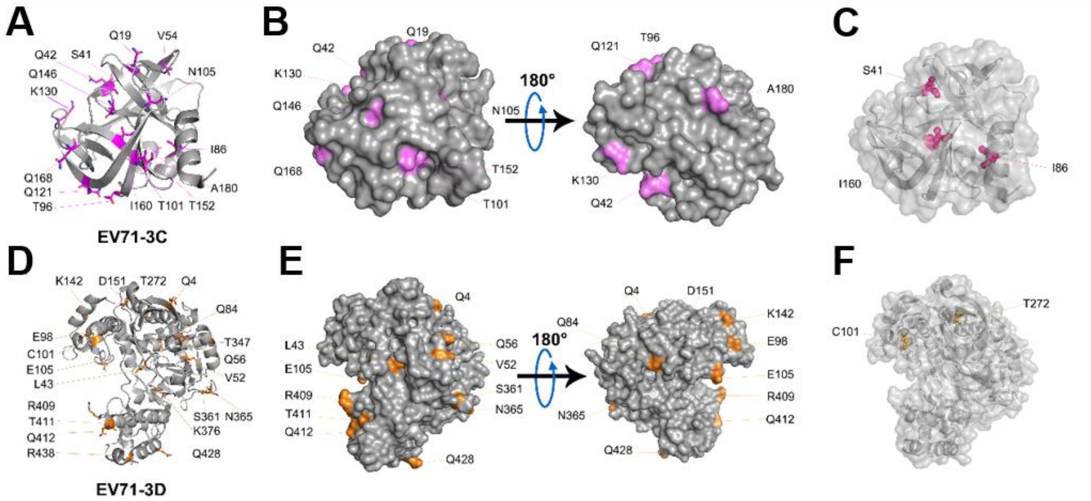

Figure S7. Structural illustration of 35 residues on EV71 3C/D. (A) Residue location on EV71 3C protein (PDB: 4GHQ). **(B)** Residues on 3C surface are highlighted in magenta. **(C)** Residues located within 3C are shown in the sphere. **(D)** Residue location on EV71 3D protein (PDB: 3N6L). **(E)** Residues on 3D surface are highlighted in orange. **(F)** Residues located within 3D are shown in the sphere.

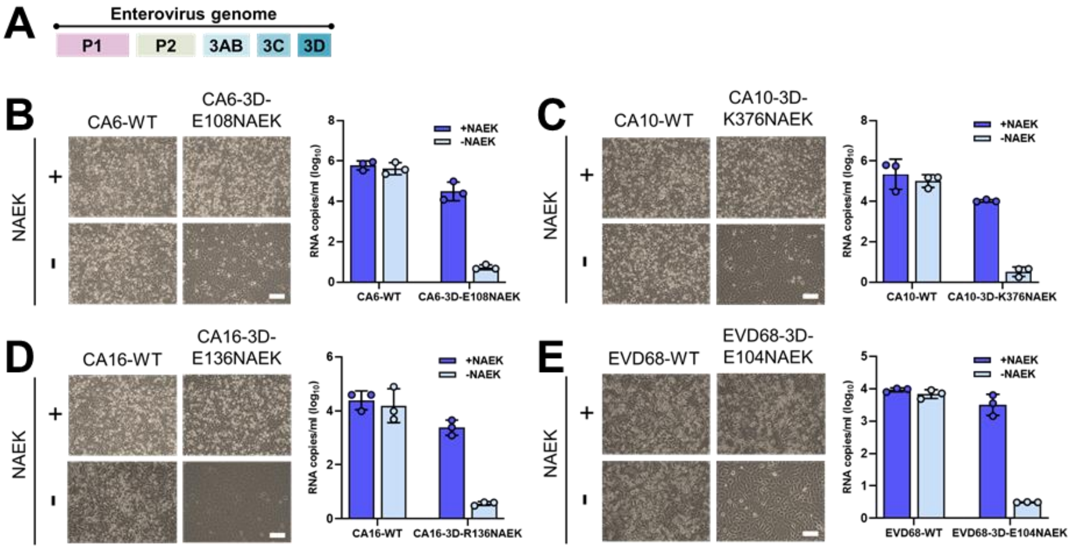

Figure S8. (A) Structure of the *Enterovirus* genome. **(B-E)** UAA-dependent CPE formation and RNA copies of CA6-3D-E108NAEK, CA10-3D-K376NAEK, CA16-3D-E136NAEK, and EVD68-3D-E104NAEK.

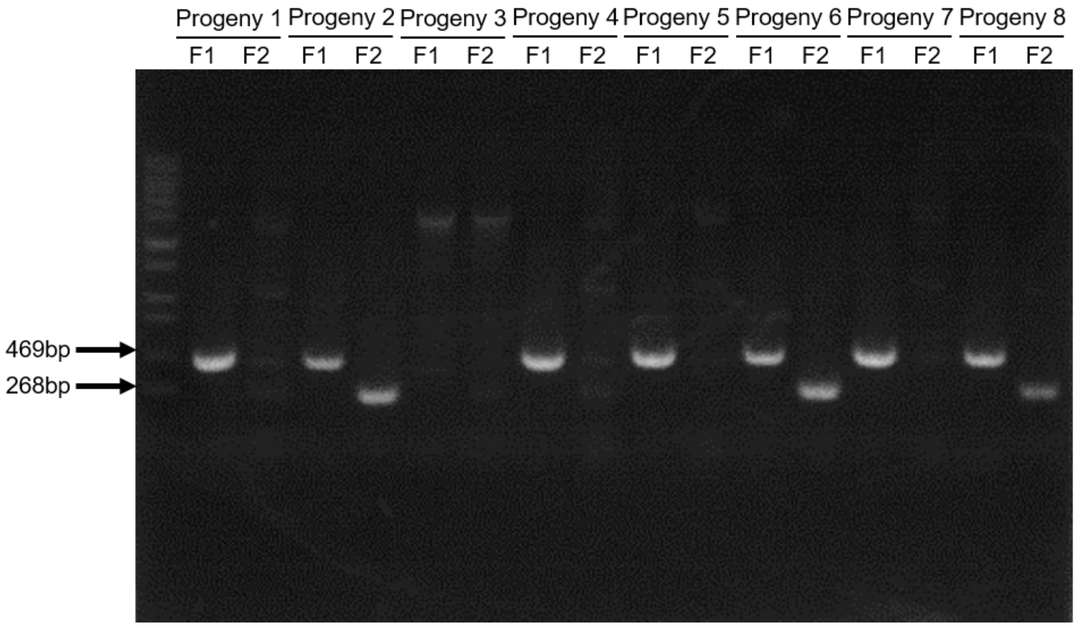

Figure S9. PCR analysis of mouse tail DNA from six descendants of transgenic mice crossed with wild-type mice. We used two primers; Primer 1 targeted the sequence at 900 bp, Primer 2 at 300 bp. The six descendants were from the F1 generation. Results showed that descendants 5 and 6 were positive for effective PylRS-tRNA$^{Pyl}_{CUA}$ pairs integration.

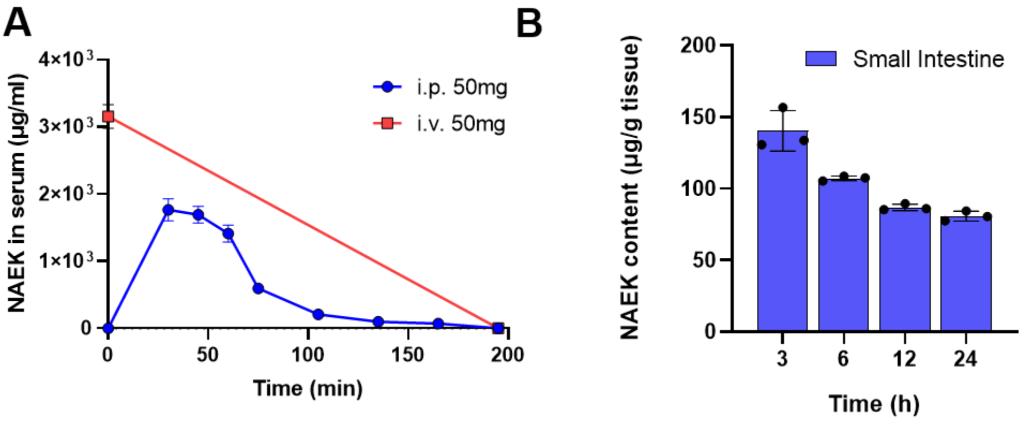

Figure S10. Bioavailability of NAEK in mice. Bioavailability of NAEK (administered i.p. and i.v.) in **(A)** serum and **(B)** tissue (small intestine) was as estimated by AUC. One 500-mg dose of NAEK was administered. Data are presented as mean ± SD (n = 3)

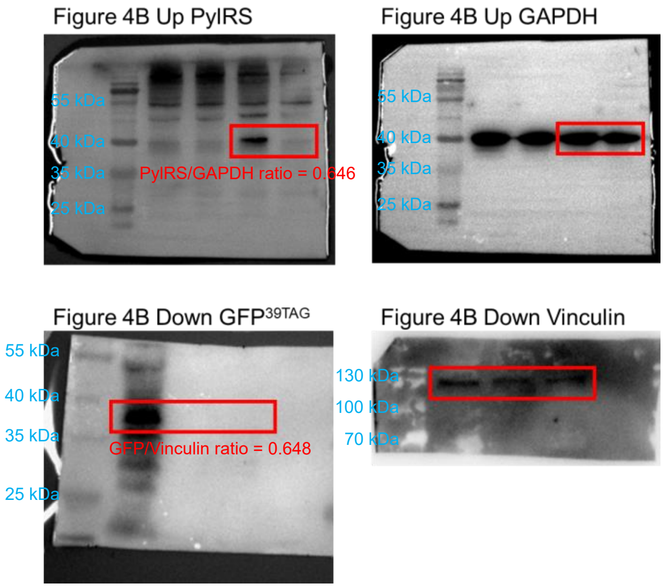

Figure S11. Original western blot images.
